# ncRNA-disease association prediction based on sequence information and tripartite network

**DOI:** 10.1186/s12918-018-0527-4

**Published:** 2018-04-11

**Authors:** Takuya Mori, Hayliang Ngouv, Morihiro Hayashida, Tatsuya Akutsu, Jose C. Nacher

**Affiliations:** 1 Department of Information Science, Toho University, Miyama 2-2-1, Funabashi, Chiba, 274-8510 Japan; 2 Bioinformatics Center, Institute for Chemical Research, Kyoto University, Gokasho, Uji, Kyoto, 611-0011 Japan; 3 Department of Electrical Engineering, Matsue College of Technology, Matsue, 690-8518 Japan

**Keywords:** ncRNA-disease association predictions, Tripartite network, Resource allocation

## Abstract

**Background:**

Current technology has demonstrated that mutation and deregulation of non-coding RNAs (ncRNAs) are associated with diverse human diseases and important biological processes. Therefore, developing a novel computational method for predicting potential ncRNA-disease associations could benefit pathologists in understanding the correlation between ncRNAs and disease diagnosis, treatment, and prevention. However, only a few studies have investigated these associations in pathogenesis**.**

**Results:**

This study utilizes a disease-target-ncRNA tripartite network, and computes prediction scores between each disease-ncRNA pair by integrating biological information derived from pairwise similarity based upon sequence expressions with weights obtained from a multi-layer resource allocation technique. Our proposed algorithm was evaluated based on a 5-fold-cross-validation with optimal kernel parameter tuning. In addition, we achieved an average AUC that varies from 0.75 without link cut to 0.57 with link cut methods, which outperforms a previous method using the same evaluation methodology. Furthermore, the algorithm predicted 23 ncRNA-disease associations supported by other independent biological experimental studies.

**Conclusions:**

Taken together, these results demonstrate the capability and accuracy of predicting further biological significant associations between ncRNAs and diseases and highlight the importance of adding biological sequence information to enhance predictions.

## Background

Recent studies have investigated the biological functions, transcriptome, and regulation of non-coding RNAs (ncRNAs) of all sizes in a wide range of organisms [1]. siRNA (short interfering RNA), miRNA (microRNA) and piRNA (piwi-interacting RNA) are the three main types of short ncRNAs (less than 30 nucleotides). They play an important role in histone modification, gene silencing, heterochromatin formation, and DNA methylation, targeting at the transcriptional and post-transcriptional levels. Long non-coding RNAs (lncRNAs), which are greater than 200 nucleotides, are relevant in fundamental processes of gene regulation, such as chromatin modification and transcriptional regulation [2]. Studies indicate that lncRNAs can be categorized in one or more of the four archetypes, which include signal archetype (molecular signal or transcriptional activity indicator), decoy archetype (measure and adjust the balance of RNA regulation), guide archetype (directs the localization of ribonucleoprotein complexes to their targets), and scaffold archetype (a structural role for relevant proteins or RNAs to resemble). However, the main functions, structures, and mechanisms of lncRNAs remain unknown [3].

Because of their categorization into the four archetypes, ncRNAs have been proposed to have strong connections with the development and pathophysiology of diseases. Computational and experimental studies have demonstrated that alteration and deregulation of both short ncRNAs and lncRNAs at the structural and expression levels, can cause various types of cancer, such as breast cancer, leukemia, hepatocellular, and colon cancer, as well as neurodegenerative disorders, cardiovascular diseases, and immune-mediated diseases [4].

X-inactive specific transcript (Xist) is a lncRNA located on the X chromosome of the placental mammals that plays an important role in the X inactivation process. Lee et al. [5] demonstrated that Xist is a potent suppressor of hematological cancer in mice. Xist is required for hematopoietic stem cell survival and function. Therefore, Xist deletion results in leukemia, marrow fibrosis, and histiocytic sarcoma.

Yang et al. [6] also demonstrated a strong correlation between lncRNAs in tumor tissues and hepatocellular carcinoma (HCC). According to their experimental results, Cox regression analysis showed that both lncRNAs H19 and UCA1 were serious risk factors for HCC. Furthermore, logistic regression also indicated that H19 was overexpressed in hepatitis B virus-infected individuals.

A study on lncRNA PRINS (Psoriasis susceptibility-related RNA Gene Induced by Stress) provides further evidence on an association between ncRNAs and diseases. PRINS is transcribed by RNA polymerase II and expressed at various levels of human tissues [7]. PRINS is believed to play a role in susceptibility to psoriasis and has been linked to autoimmune diseases [8].

These findings are representative of the small number of ncRNA-disease connections that have been functionally established. This poses a major obstacle for bio-physicians in their quest to formulate new hypotheses for molecular mechanisms underlying complexes diseases, and to enhance the efficacy and efficiency of disease diagnosis and treatment. For the purpose of determining an association, bio-physicians must partition patients into appropriate groups and accurately investigate them. This method indeed establishes the correlation and expands the acknowledgement of the association in various phenomena. Although it is necessary to predict and infer ncRNA-disease associations, it is an incredible cost and time burden for bio-physicians.

To address this problem, some computational models have been proposed for ncRNA-disease association inference. Chen et al. [9] introduced Laplacian Regularized Least Squares for LncRNA-Disease Association (LRLSLDA), a semi-supervised learning method based on the framework of Laplacian Regularized Least Square and the assumption that ncRNAs with similar functions tend to interact with similar diseases. LRLSLDA can predict ncRNA-disease associations reliably. Yet, there are obstacles with parameter selection and classifier combinations. Another method called RWRLncD has been proposed by Sun et al. [10] that infers ncRNA-disease associations by integrating ncRNAs functional similarity network, disease similarity network, and known ncRNA-disease associations. RWRLncD cannot be employed without any known ncRNA-disease associations. Li et al. [11] introduced a genomic location-based computational method for ncRNA-disease association prediction. However, the approach has not been evaluated with statistical tests, and not all ncRNAs were associated with their neighbor genes, a major limitation of the method. Yang et al. [12] introduced a method employing a bipartite network with resource-allocation technique to infer new ncRNA-disease connections. Their experiment to validate the performance of their method mainly focused on only one dataset collected from Chen et al. (2013), with roughly 1028 interactions between 322 ncRNAs and 221 diseases. They also included additional interactions via deep literature mining. Alaimo et al. then proposed a new ncRNA-disease association prediction method called ncPred, which was shown to outperform Yang et al.’s method [13]. ncPred is a resource-propagation-based method applied on an ncRNA-target-disease tripartite network. The tripartite network, formed up by ncRNA-target and target-disease interaction bipartite networks, guided the resource transferring process to infer new associations. Targets refer to a group of genes, microRNAs or proteins that are related to particular ncRNAs in terms of expression regulation and binding activities etc. With inclusion of the targets, ncPred was experimentally shown to infer more biological information with higher reliability than Yang et al.’s method. Yet, there is still some room for improvement, particularly with regards to establishing the biological significance of identified associations. Chen et al. [14] developed hypergeometric distribution for lncRNA-disease association inference (HGLDA) to predict lncRNA-disease associations by integrating miRNA-disease interactions and lncRNA-miRNA associations. HGLDA applied *p*-value matrices obtained from interaction networks, with false discovery rate (FDR) correction. lncRNA-disease pairs with FDR less than 0.05 were predicted to be potential lncRNA-disease associations. However, HGLDA provided the least biological information, which led to weaker biological significance reliability.

Here, we introduce a method that improves on Alaimo et al.’s ncPred. The proposed method integrates a resource-allocation technique in the ncRNA-target-disease tripartite network, with pairwise similarity information obtained from sequence expressions. The prior knowledge combined with the biological information is carried and transferred throughout the network, and finally utilized to infer new associations.

To validate the performance of our proposed method, we conducted a 5-fold-cross-validation procedure to demonstrate its reliability, accuracy and efficiency, on a dataset reconstructed from Chen et al. (2013) [15]. In addition, by using a database of experimentally confirmed interactions between ncRNAs and miRNAs shown in Helwak et al. [16], we performed another validation of our proposed method. Our results demonstrate that our proposed method outperforms ncPred.

## Methods

### Data sources

**Fig. 1:**
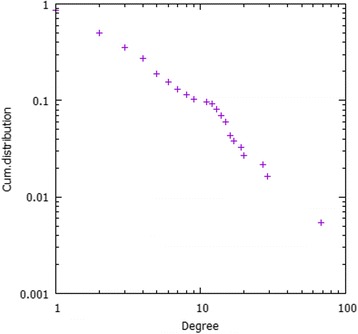
Cumulative degree distribution of the tripartite ncRNA-target disease network for Chen et al. dataset

In order to evaluate the performance of the approach, we prepared two kinds of data, ncRNA-target interaction matrix (LncRNADisease database (2015)) and target-disease interaction matrix (DisGeNET database) which are the same database sources used in Chen et al. (2013) [15], to form the tripartite network, as well as the sequence expression of those targets and ncRNAs (extracted from Uniprot and LncRNADisease databases, respectively). Since there are ncRNA sequence expressions and targets that are still unknown, we could only collect 76 ncRNAs, 109 targets, and 514 diseases (see Table 1). In Fig. 1, the degree distribution of the resulting network is shown. The results indicate that the entire network may follow a power law distribution. Moreover, we collected experimentally confirmed interactions between ncRNAs and miRNAs from the database shown in [16] and from Alaimo [13] Supplementary Information files and reconstructed another dataset composed on 151 ncNRAs, 179 targets and 134 diseases (see Helwak dataset in Table 1).

**Table 1:** Description of the datasets

Type of Data	Chen et al	Helwak et al.
Diseases	514	134
Targets	109	179
ncRNAs	76	151
Disease-target interactions	580	1572
Target-ncRNA interactions	111	610
Average degree	1.977	9.405

### Computational approach

**Fig. 2:**
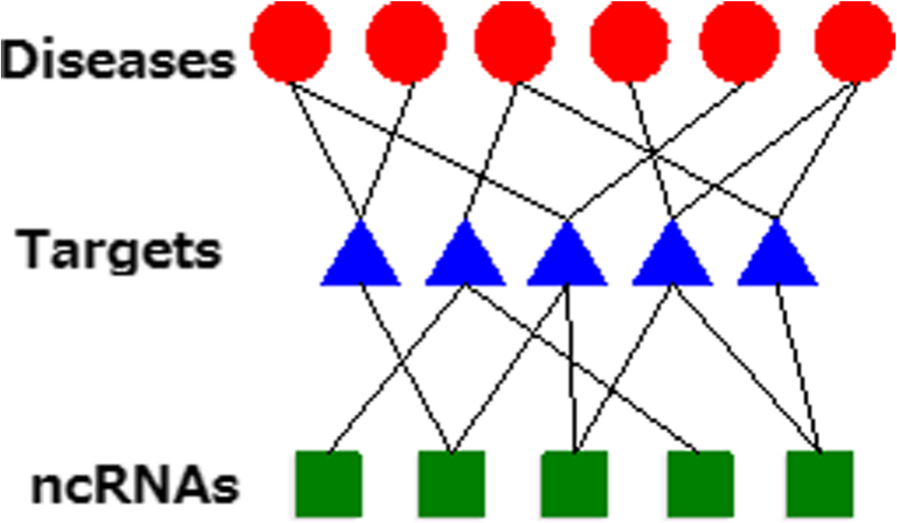
Example of a tripartite network used in this work. Targets integrate information from ncRNAs and diseases

**Fig. 3:**
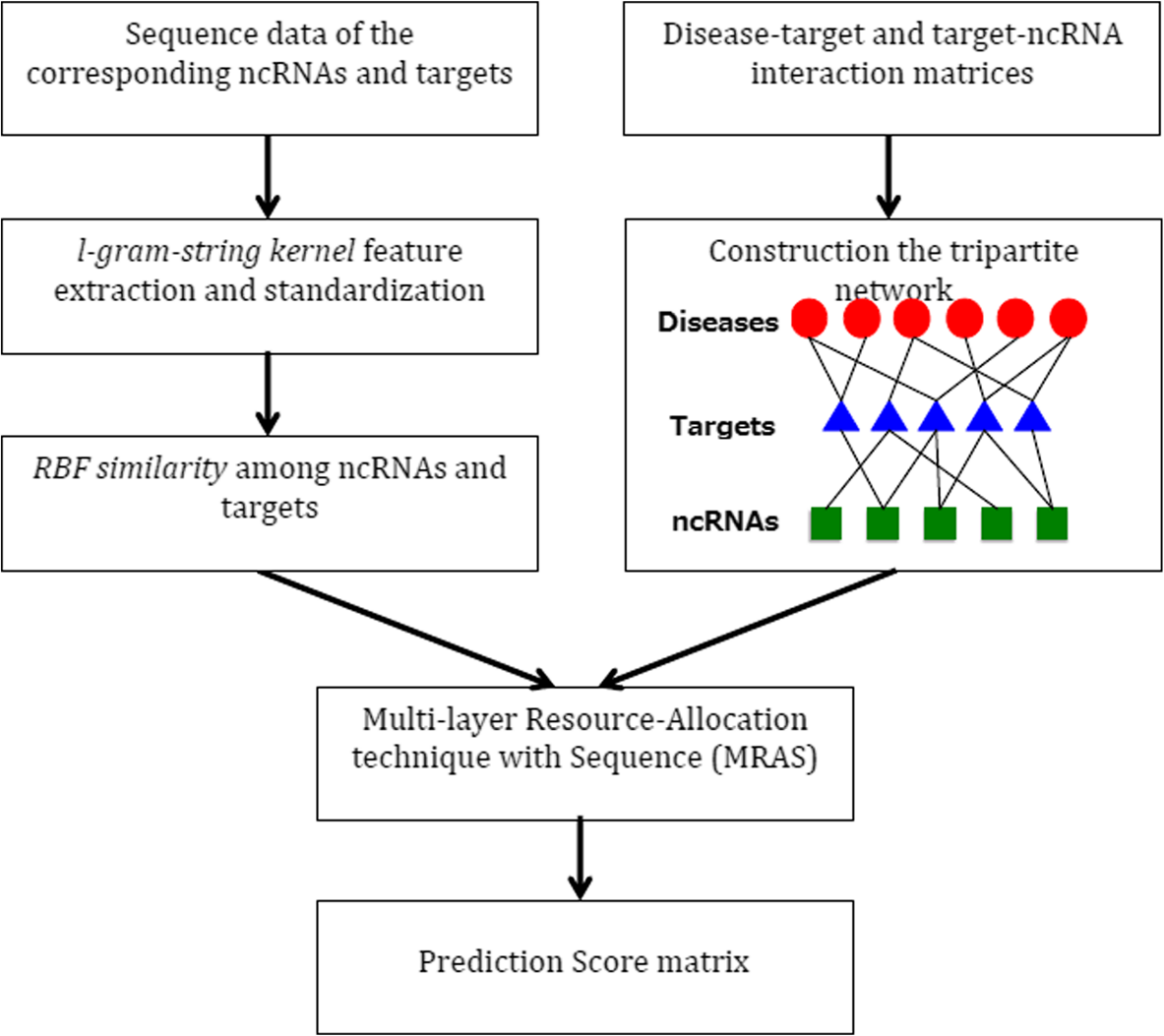
Illustration of the proposed computational method. Sequence information analysis is combined with a tripartite network structure. A multi-layer resource-allocation technique integrates the information and predicts associations between ncRNAs and human diseases

In our approach, disease-target and target-ncRNA interaction matrices are required to construct the tripartite network (See Fig. 2). Sequence expressions of both targets and ncRNAs are also needed to generate string-kernel features for the prediction method. The overall algorithm of our method is shown in Fig. 3.

Let *D* = {*d*_1_, *d*_2_, …, *d*_*m*_} be a set of diseases, let *T* = {*t*_1_, *t*_2_, …, *t*_*n*_} be a set of targets which refer to genes or microRNA, and let *R* = {*r*_1,_*r*_2_, …, *r*_*p*_} be a set of non-coding RNAs. Let ${SR}^l=\left\{{sr}_1^l,{sr}_2^l,\kern1.5em ,{sr}_p^l\right\}$ and let ${ST}^l=\left\{{st}_1^l,{st}_2^l,\dots, {st}_n^l\right\}$ be sets of the sequence expressions of the targets and ncRNAs respectively.

### L-gram-string kernel feature extraction and standardization

In our approach, the features of sequence expressions were extracted via the *l-gram-string* kernel method [17], in which each string is transformed into a vector consisting of the number of occurrences of each substring of length *l*. In bioinformatics, string kernel is considered a function that measures the similarity of a pair of sequence expressions (strings) with finite length, for the purpose of generating real-value feature vectors. In our case, the method was evaluated experimentally using length *l* = 1, 2, 3 and 4.

In order to improve the quality and reduce the redundancy of the computed features from the previous step, the standardization method below was applied [18].

$$ {x}^{\prime }=\frac{x-\overline{x}}{\sigma }, $$

where *x* is the original feature vector, $\overline{x}$ is the mean and *σ* is the standard deviation.

Thus, let ${SST}^l=\left\{{sst}_1^l,{sst}_2^l,\dots, {sst}_n^l\right\}$ and ${SSR}^l=\left\{{ssr}_1^l,{ssr}_2^l,\dots, {ssr}_p^l\right\}$ be sets of the normalized real-value feature vectors computed from the sequence expressions of the targets and ncRNAs respectively.

### *RBF kernel similarity* among ncRNAs and targets

To effectively compute the similarity among each pair of ncRNA-ncRNA and target-target, the RBF kernel similarity technique was employed. In statistical learning, RBF kernel can be viewed as a common kernel function to measure the similarity [19].

Let *k*_*rna* (*i*, *j*) and *k*_*target* (*i*, *j*) be the RBF kernel similarity function of pair ncRNA-ncRNA and target-target respectively.

Thus, the RBF similarity of *i*^*th*^ ncRNA and *j*^*th*^ ncRNA can be computed as

$$ k\_ rna\ \left(i,j\right)=\exp \left(-{\gamma}_1{\left\Vert {ssr}_i^l-{ssr}_j^l\right\Vert}^2\right), $$

where $$ {\gamma}_1=\frac{1}{2{\sigma}_1^2},{\sigma}_1\ \mathrm{is}\ \mathrm{the}\ \mathrm{parameter}\ 0<{\sigma}_1<1. $$

The RBF similarity of *i*^*th*^ target and *j*^*th*^ target can be computed as

$$ k\_ target\ \left(i,j\right)=\exp \left(-{\gamma}_2{\left\Vert {sst}_i^l-{sst}_j^l\right\Vert}^2\right), $$

where $$ {\gamma}_2=\frac{1}{2{\sigma}_2^2},{\sigma}_2\ \mathrm{is}\ \mathrm{the}\ \mathrm{parameter}\ 0<{\sigma}_2<1. $$

### Constructing the tripartite network disease-target-ncRNA

*G (D, T, R, E)*, where *E* denotes a set of edges, is a tripartite network (Fig. 2) representing the disease-target interactions and target-ncRNA interactions. ${A}^{DT}={\left\{{a}_{ij}^{DT}\right\}}_{m\times n}$ denotes the adjacency matrix of the disease-target network, and ${A}^{TR}={\left\{{a}_{ij}^{TR}\right\}}_{n\times p}$ represents the adjacency matrix of target-ncRNA network.

Targets represent a group of biomolecules functionalized by non-coding RNAs. Targets can be genes, proteins, microRNAs, etc., whose activities include expression regulation, binding, and complex formation. In our method, targets function as a bridge allowing us to extract further biological information, for enhancing the accuracy of disease-ncRNA association inference.

Our method integrates the resource-allocation algorithm in the network with pairwise similarity information derived from sequence expressions to produce scores showing the level of certainty of the interaction. Essentially, the resource-allocation algorithm carries prior understanding of the bipartite network, which can be employed to predict the interactions.

### Multi-layer resource-allocation technique with sequence (MRAS) information

Since the tripartite network consists of two bipartite networks, two-layer resource-allocation techniques were applied. For the first-layer (disease-target bipartite network), the resource will be transferred from the nodes in *T* (targets) to the nodes in *D* (diseases) integrating with pairwise similarity information between each pair of the diseases, then move back to the nodes in *T*. For the second-layer (target-ncRNA bipartite network), the resource integrated with pairwise similarity information between each pair of the ncRNAs, is allocated from the nodes in *R* (ncRNAs) to the nodes in *T*, and then combined with the resource from the previous layer. Finally, the weights computed within the two layers were merged into one, called combined weight (*W*_*C*_), indicating the likelihood that in case a disease associates with a target *t*_*i*_, it then possibly interacts with ncRNA *r*_*j*_. Prediction scores (*P*) can then be computed from the combined weight *W*_*C*_, and the higher the score, the greater the certainty that the ncRNA will associate with a particular disease.

Let deg(*x*) be the degree of node *x* in the disease-target network, and deg^′^(*y*) be the degree of node *y* in the target-ncRNA network.

- Layer 1: disease-target bipartite network
  Let ${W}^T={\left\{{w}_{ij}^T\right\}}_{n\times n}$ be the probability that the *i*^*th*^ target interacts with the *j*^*th*^ target when both of them interact with the same disease:

$$ {w}_{ij}^T=k\_ target\left(i,j\right)\cdot \sum \limits_{l= 1}^m\frac{a_{li}^{DT}{a}_{lj}^{DT}}{\mathit{\deg}\left({d}_l\right)} $$

- Layer 2: target-ncRNA bipartite network
  Let ${W}^R={\left\{{w}_{ij}^R\right\}}_{p\times p}$ be the probability that the *i*^*th*^ ncRNA interacts with the *j*^*th*^ ncRNA when both of them interact with the same target:

$$ {w}_{ij}^R=k\_ rna\left(i,j\right)\cdot \sum \limits_{l=1}^n\frac{a_{li}^{TR}{a}_{lj}^{TR}}{\deg \left({t}_l\right)} $$

Hence, the combined weight ${W}_C={\left\{{w}_{ij}^C\right\}}_{n\times p}$ of the two bipartite networks together with the similar neighborhood of both targets and ncRNAs can be obtained as follows. The formula assigned more weight to the path with higher frequency.

$$ {w}_{ij}^C=\sum \limits_{t=1}^n\left[{w}_{it}^T\sum \limits_{r=1}^p\left({a}_{tr}^{TR}\cdot {w}_{rj}^R\right)\right] $$

Finally, the prediction score matrix can be computed based on the following formula: *P* = {*p*_*ij*_}_*m* × *p*_ = *A*^*DT*^ ⋅ *W*_*C*_

Based on the above procedures, the prediction score matrix was produced via the integration of biological similarities of sequence expressions and the resource-allocation technique in the tripartite network. The score matrix thus predicted associations between ncRNAs and diseases. The higher the score, the higher the certainty of ncRNA-disease connectivity.

## Results

### Algorithm prediction performance and evaluation

**Fig. 4:**
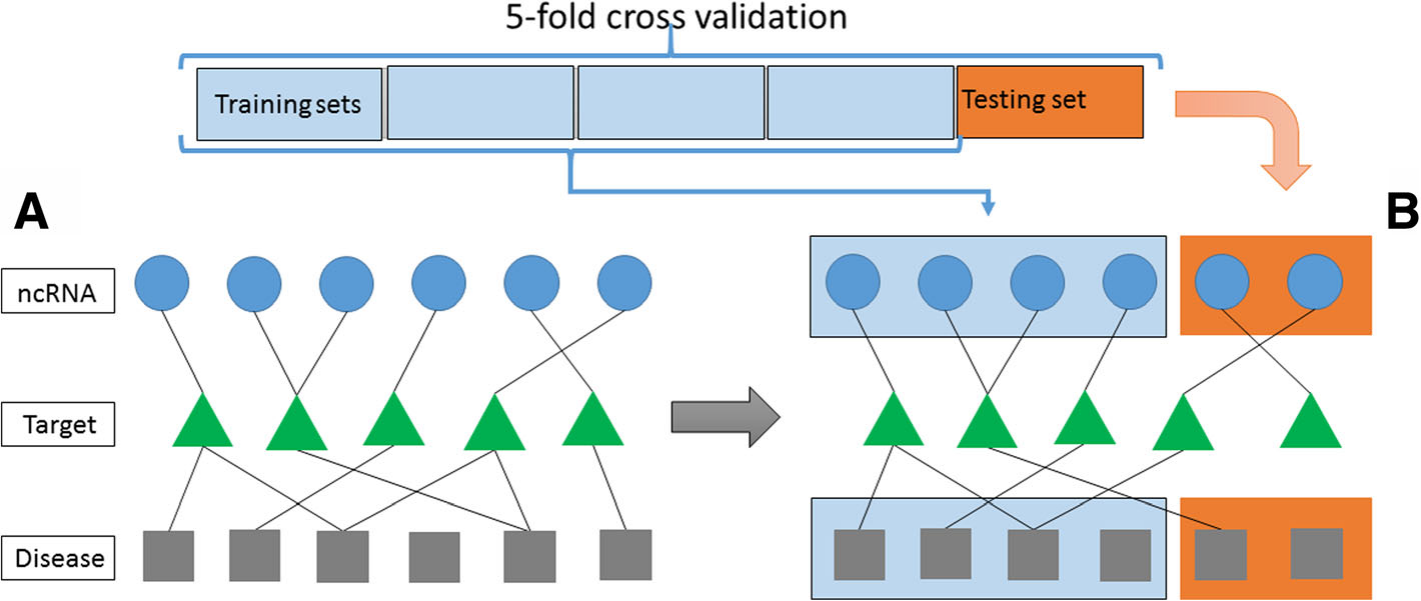
Algorithm performance evaluation methodology using 5-fold cross validation. From the constructed network as shown in (**a**), we divide all ncRNA-disease pairs into 5 groups. Each time one group performs as a testing dataset. To evaluate whether the algorithm is able to predict the interacting pairs without memory of existing interactions, we delete links that connect ncRNAs with disease if they belong to the testing set as shown in (**b**)

As mentioned, the performance of our method was measured by applying a 5-fold-cross-validation procedure as well as other computational operations. The evaluation algorithm is described as follows:

1. First, we consider all pairs of ncRNA and disease (*N* pairs) exiting in the tripartite network. These pairs are expected to be predicted by the algorithm in the score matrix *P*.
2. Fix a pair of values for *σ*_1_ and *σ*_2_ .
3. Consider k-fold cross validation with *k = 5*. Therefore, all *N* pairs are divided into 5 groups.
4. Each group of *N/5* elements becomes a test data one time. The rest four groups are training data
5. From each test data group, we scan those pairs that are connected through targets and disconnect them (see Fig. 4b)
6. Run the algorithm and make predictions only for the pairs in the test group *P*_*test.*_
7. Construct a ROC curve for the test group based on *N/5* scores for *P*_*test*_.
8. Repeat 3~ 7 for different folds. We average all five ROC curves.
9. Repeat 3~ 8 for randomization of test and training groups five more times and obtained an averaged ROC curve.
10. Repeat 2~ 9 for each *σ*_1_ [0.1,0.2,...,0.9] x *σ*_2_ [0.1,0.2,..,0.9] and pick up the best AUROC among all results.

### Evaluation results

We took into account the area under receiver operating characteristic (ROC) curve (AUC) values to assess the reliability, credibility, and accuracy of the prediction method. Table 2 demonstrates that our proposed method clearly outperforms ncPred in terms of the average values of AUC for the analysed datasets as shown in Table 1. These results emphasize the improved reliability and accuracy of predicting new ncRNA-disease associations or biological relevance using our approach, compared to that of Alaimo et al. which uses ncPred.

**Table 2:** Comparison of the proposed method and ncPred through averaged area under ROC curve (ROC)

AUC		
	Proposed method	ncPred
Chen et al.(2013)	*0.57135*	0.50242
Helwak et al.(2013)	*0.77444*	0.50295

Italic numbers indicate the best performance

**Fig. 5:**
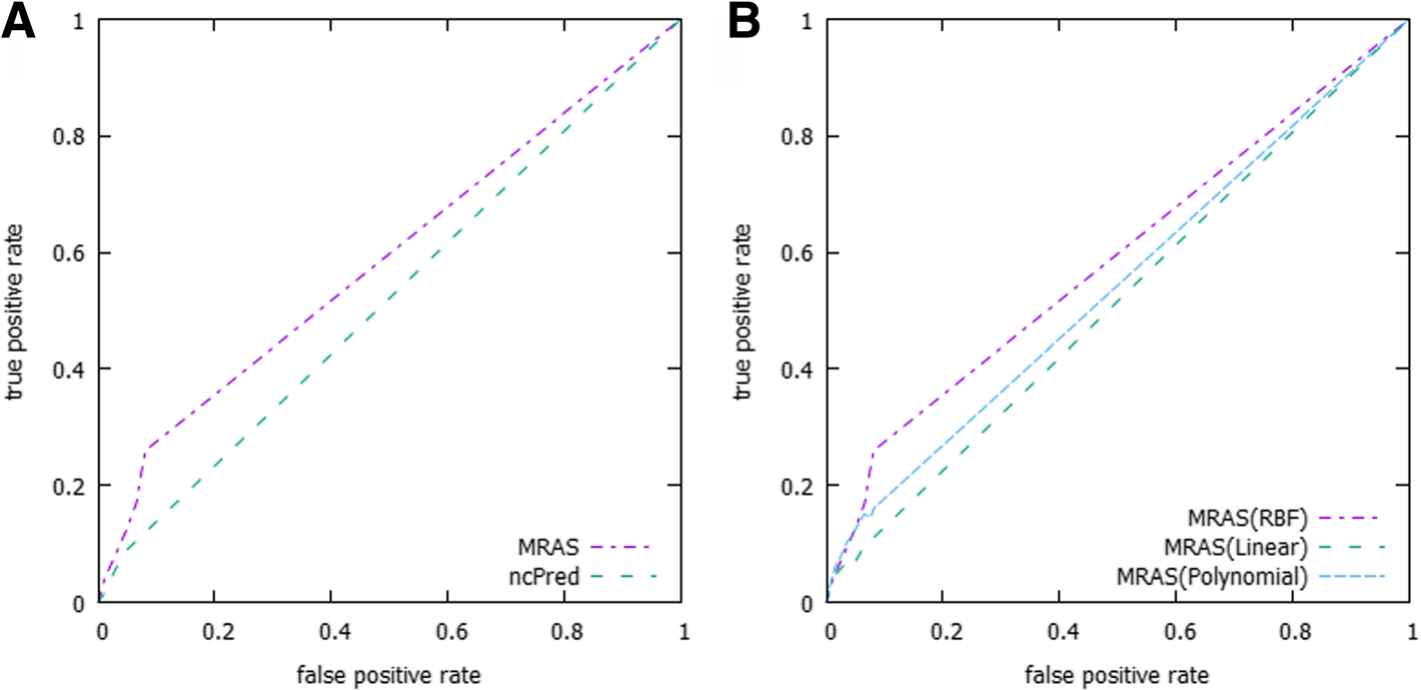
Comparison between the proposed method (MRAS) and ncPred. **a** shows an ROC curves comparison between the proposed method (MRAS) and ncPred with evaluation of the Chen et al. dataset. The ROC curves were drawn up based on the average results of the simulation, and repeated to ensure reliable estimates as described in the evaluation method procedure. The results also convey that the proposed method produced a higher true positive rate, which demonstrates its superior performance over its competitor. **b** shows the results of the proposed method using different kernel functions

In our approach, the RBF kernel technique was selected to conduct the similarity measure. In order to prove that RBF kernel can interpret the similarity of biological significance more appropriately, we conducted comparison experiments on the dataset listed in Table 1 with three representative kernel functions: linear kernel, polynomial (degree = 2) kernel and RBF kernel. Table 3 clearly illustrates the prediction performance of our approach underlying each kernel function. The results demonstrate that RBF kernel successfully outperforms the other two kernel techniques, showing the highest average AUC values (see Fig. 5b).

**Table 3:** Comparison of performance using three representative kernel functions

Dataset	AUC		
	Linear Kernel	Polynomial Kernel (d = 2)	RBF Kernel
Chen et al. (2013)	0.51561	0.54175	**0.57135**

Performance results for the following three kernel functions: linear kernel, polynomial (degree = 2) kernel and RBF kernel. The length of *l-gram* is set to *l = 3* in this experiment

**Fig. 6:**
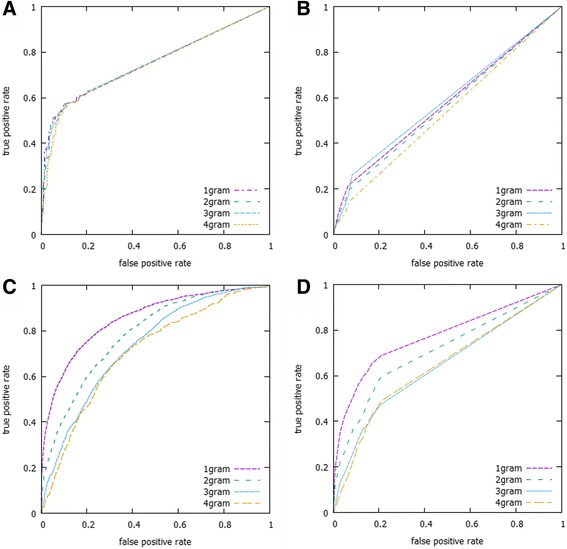
Illustration of the ROC curves for the proposed algorithm**.** ROC curves for the proposed algorithm MRAS using different values of *l-*gram string (*l =* 1, 2, 3 and 4) and using RBF kernel (**a**) without link cut (AUC = 0.75) and (**b**) with link cut (AUC = 0.57) for the Chen dataset. The results for the Helwak dataset using RBF kernel (**c**) without link cut (AUC = 0.85) and (**d**) with link cut (AUC = 0.77)

The *l-gram-string kernel* technique was applied to extract biological features from the sequence data of ncRNAs and targets in the proposed method. In order to investigate which length of *l* can interpret and extract better information from the sequence data, we validated the performances underlying different lengths of *l*. We also compared the results of the proposed method under two assumptions. By using the cut link method described in algorithm performance evaluation section and without cut link. Table 4 shows the comparison for both methods and for *l-gram-string kernel* (when *l* = 1, 2, 3 and 4) in terms of AUC values using the dataset described in Table 1. The results show that link cut notably decreases the prediction of the algorithm as expected from 0.75 to 0.57 (see Fig. 6a and b). However, even though we perform this strict procedure, the algorithm is able to predict correctly interactions with an AUC above 0.5. The results also show that performance slightly improved at *l* = 3. Thus, *l* = 3 is the optimal parameter for the method (see Table 4 and Fig. 6b). Note that for Helwak dataset the optimal length is *l = 1.*

**Table 4:** Comparison of *l-gram-string kernel* in terms of average Area Under the Curve (AUC) values

		*l = 1*	*l = 2*	*l = 3*	*l = 4*
Chen et al.(2013)	**AUC**	0.7507	0.7506	*0.7513*	0.7433
	**Cut link AUC**	0.5706	0.5644	*0.5713*	0.5476
Helwak et al.(2013)	**AUC**	*0.8543*	0.7855	0.7338	0.7080
	**Cut link AUC**	*0.7744*	0.7098	0.6430	0.6441

The performance of the *l-gram-string kernel* for *l* = 1, 2, 3 and 4 was examined using the AUC metric. The computation was done using the RBF kernel. Table also shows the prediction performance when the links between a disease and their associated ncRNAs are deleted (Cut link UAC). Italic numbers indicate the best performance

For parameter tuning of RBF kernel similarity function *σ*_1_ and *σ*_2_ we selected the optimal parameters as explained in the evaluation method procedure. Table 5 shows the optimal values identified in our computation.

**Table 5:** Optimal values of *σ*_1_ and *σ*_2_ parameters

	*σ*_1_	*σ*_2_
Chen et al.(2013)	0.6	0.7
Helwak et al.(2013)	0.9	0.1

### Case studies

In order to demonstrate the credibility and functionality of the proposed method, we compared predicted disease-ncRNA pairs using Chen et al. dataset with recent experimental studies, as shown in Table 6, on six diseases, namely lung cancer, liver cancer, breast cancer, urinary bladder neoplasms, prostatic neoplasms and stomach neoplasms**.**

**Table 6:** The predicted ncRNAs related to the six diseases examined in this study

Disease	ncRNA	Evidence (PMID)
Breast Neoplasms	H19	15985428;27540977;9811352;24780616
Breast Neoplasms	PVT1	17908964;10485452;23907597
Breast Neoplasms	SRA1	17710122;26460974
Breast Neoplasms	WRAP53	27525856;15736456
Carcinoma, Hepatocellular	H19	9570364
Carcinoma, Hepatocellular	PCNA-AS1	24704293
Carcinoma, Hepatocellular	WRAP53	23836507
Lung Neoplasms	HOTAIR	25010625;22088988
Lung Neoplasms	MALAT1	23243023;26490983
Lung Neoplasms	PVT1	25400777
Lung Neoplasms	XIST	26339353
Prostatic Neoplasms	CDKN2B-AS1	27197191;24988946
Prostatic Neoplasms	H19	19513555;25895025
Prostatic Neoplasms	HOTAIR	26411689
Prostatic Neoplasms	MALAT1	23845456
Prostatic Neoplasms	PCAT1	21804560
Prostatic Neoplasms	SRA1	16607388
Prostatic Neoplasms	XIST	16261845
Stomach Neoplasms	CDKN2B-AS1	24810364;26187665
Stomach Neoplasms	HOTAIR	25063030;23898077
Stomach Neoplasms	MALAT1	24857172
Urinary Bladder Neoplasms	H19	23354591
Urinary Bladder Neoplasms	WRAP53	27912092

The predicted ncRNAs related to lung cancer, liver cancer, breast cancer, urinary bladder neoplasms, prostatic neoplasms and stomach neoplasms based upon our method. Each research article reporting on a specific ncRNA is shown with a unique ID (*PMID*) by PubMed

### Lung cancer

Lung Cancer (LC) is one of the most deadly diseases affecting both men and women worldwide. There are three types of lung cancer: non-small cell lung cancer, small cell lung cancer, and lung carcinoid tumor. In 2015 lung and bronchus cancer accounted for 13.3% of all cancer cases in the US. The proposed method predicted that seven ncRNAs, H19, HOTAIR, MALAT1, PVT1, WRAP53, XIST, CDKN2B-AS1 are associated with LC. Kondo et al. reported that the alteration and deregulation of H19 impacts lung cancer cell growth [20]. HOX transcript antisense RNA (HOTAIR) prevents gene expression via collection of chromatin modifiers. Loewen et al. demonstrated that HOTAIR plays an important role in the intervention of lung cancer [21]. lncRNA metastasis associated lung adenocarcinoma transcript 1 (MALAT1) can impair in vitro cell motility of lung cancer cells and simultaneously influence a number of genes (Tano et al. & Tseng et al. [22, 23]). Yang et al. reported that an increased expression of the lncRNA PVT1 promotes tumorigenesis in non-small cell lung cancer [24]. Tantai et al. suggested a combined identification of long non-coding RNA XIST and HIF1A-AS1 in serum as an effective screening for non-small cell lung cancer [25]. Park et al., observed evidences for lung cancer with two variants located in cancer pleiotropic regions, namely *TERT* and risk of lung adenocarcinoma and *CDKN2BAS1* with risk of lung squamous cell carcinoma [26].

### Liver cancer

Liver cancer is the third most deadly cancer worldwide [27]. Very few patients receive curative treatments, while the majority do not recover since they are diagnosed at later stages. Our method predicted that ncRNAs H19, PCNA-AS1 and WRAP51 are correlated with liver cancer. H19 ncRNA expression was shown to result in high H19 protein expression in liver cancer whenever there is a loss of imprinting [28]. Iizuka et al. investigated further the epigenetic abnormalities in the insulin-like growth factor 2 (IGF2) and H19 genes observed in hepatocellular carcinoma (HCC) [29]. Yuan et al. reported that antisense long non-coding RNA PCNA-AS1 promotes tumor growth in hepatocellular carcinoma [30]. Several studies have also linked WRAP51 with HCC [31].

### Breast cancer

Breast cancer is a ductal carcinoma beginning in the cells of lobules, ducts and other tissues of the breast. In the United States, breast cancer is the second most common cancer, just after skin cancer. Both women and men can suffer from this severe disease. Our method predicted that four ncRNAs, H19, PVT1, SRA1 and WRAP53 may be associated with this disease. Berteaux et al. found out that H19 transcript antisense RNA stabilize breast cancer cells and is overexpressed in breast tumors [32]. Others evidences were found across literature [33, 34]. Zhang et al. reported that the non-protein coding plasmacytoma variant translocation 1 (PVT1) has been implicated in human cancers [35, 36]. In addition, Hube et al. reported that alternative splicing of SRA1 could lead to the generation of coding and non-coding RNA isoforms in breast cancer cell lines [37]. Cao et al. recently reported about the association between the WRAP53 gene rs2287499 C > G polymorphism and cancer risk [38].

### Urinary bladder Neoplasms

Urinary bladder neoplasms are the result of abnormal growth of bladder cells, and are considered as one of the common cancers. Men are at higher risk for this disease than women. The predictive results derived from our method inferred that ncRNAs H19 and WRAP53 might be associated with urinary bladder cancer. Luo et al. demonstrated that the abnormal expression of H19, particularly its up-regulation, contributed to cell proliferation in bladder neoplasms [39]. Wrap53 is a multi-functional gene which is capable of regulating p53 levels in both normal and cancer cell lines [40].

### Prostatic Neoplasms

Prostatic neoplasm is caused by uncontrolled growth of cells located in the prostate causing tumors. Based upon our proposed method, prostate cancer was predicted to be associated with H19, MALAT1, CDKN2B-AS1, HOTAIR, PCAT1, SRA1, XIST. Zhu et al. demonstrated that H19 was significantly downregulated in the metastatic prostatic tumor cell line M12 [41]. Perez et al. showed that the antisense intronic transcript of MALAT1 is correlated with tumor differentiation in prostate cancer [42]. Fehringer reported the involvement of CDKBN2B-AS1in Cross-cancer genome-wide analysis of lung, ovary, breast, prostate and colorectal cancer using a cross-cancer genome-wide analysis [43]. Zhang et al. discovered that LncRNA HOTAIR enhances the Androgen-Receptor-Mediated Transcriptional Program and Drives Castration-Resistant Prostate Cancer [44]. Ren et al. suggested the Long noncoding RNA MALAT-1 as a potential therapeutic target for castration resistant prostate cancer [45]. Presner et al. results implied that rhe Long Non-Coding RNA PCAT-1 Promotes Prostate Cancer Cell Proliferation through cMyc [46]. The same research group used a transcriptome sequencing across a prostate cancer cohort to identify PCAT-1 as an unannotated lincRNA implicated in disease progression [47]. Several works also reported on the involvement of SRA1 [48] and XIST [49] in prostatic neoplasm.

### Stomach Neoplams

Stomach neoplams that occurs as a consequence of abnormal grothw of stomach cells have also been associated to ncRNAs in several works. Our method predicted CDKN2B-AS1, HOTAIR and MALAT1 as main correlated molecules with stomach neoplasms. Zhang et al., reported that ANRIL (CDKN2B-AS1) which recruits and binds to PRC2 is usually observed upregulated in human gastric cancer (GC) tissues [50]. Lee et al. found that long non-coding RNA HOTAIR tends to promote not only carcinogenesis but also invasion of gastric adenocarcinoma [51]. Wang et al. also observed that MALAT1 promotes cell proliferation in gastric cancer by recruiting SF2/ASF [52].

## Discussion

Our work has improved the prediction quality and performance of Alaimo et al.’s method ncPred by integrating a biological feature information derived from sequence data with weight calculated by the multi-layer resource allocation technique, throughout the whole disease-target-ncRNA tripartite network.

The proposed method is unique because involves three types of biological information, namely ncRNAs sequences, target sequences and diseases. This rich biological information is also organized in a complex tripartite network in which targets integrate information from ncRNAs and diseases. Our study extended Alaimo’s approach [13] because we integrated biological sequence information. The uniqueness of the datasets and the complexity of the networks structure make it difficult to perform a straightforward comparison of our predictions with other approaches besides ncPred algorithm [13]. Indeed, most of the previous works have been done using a more simple approach involving a bipartite network. For example, Yang el al [12] constructed a bipartite network composed of only lncRNAs and diseases. On this network, a propagation algorithm navigated to infer lncRNAs implicated in diseases. Alaimo et al. [13] have already shown that their method outperformed the method by Yang et al. [12].

Our computational analyses indicate that our approach could result in more reliable and biologically efficient disease-ncRNA associations prediction than ncPred (See Tables 2 and 6). The prediction scores were obtained based on significant biological information, which provided useful suggestions of which ncRNA-disease associations have stronger interactions. This paves an easy path for pathologists to analyze and interpret ncRNA-disease associations. However, in order to accurately determine and confirm the associations, suitable patients and document cases are still needed.

The proposed method still has limitations that need to be considered. For instance, it lacks ncRNA-target interaction data and their particular sequence data. Those sequence data are required to extract the feature vector, which could extend the computational time. Furthermore, the lengths of ncRNAs and targets vary, ranging from less than 100 nucleotides to more than 10,000 nucleotides. This impacts the ranking of predicted ncRNA-disease associations. Accordingly, more related datasets are required to improve and expand the reliability and quality of ncRNA-disease association inference.
